# Association of Plasma Omentin-1 Levels With Diabetes and Its Complications

**DOI:** 10.7759/cureus.18203

**Published:** 2021-09-23

**Authors:** Abdul Habib Eimal Latif, Sana Anwar, Kanagala Sai Gautham, Firas Kadurei, Roland Oluwapelumi Ojo, Farukhzad Hafizyar, Daniya Muhammad Haroon, FNU Rakesh, Abdul Subhan Talpur

**Affiliations:** 1 Internal Medicine, Kabul University of Medical Sciences, Kabul, AFG; 2 Internal Medicine, Lugansk State Medical University, Ferndale, USA; 3 Internal Medicine, Osmania Medical College, Hyderabad, IND; 4 Internal Medicine, Mubarak Al-Kabeer Hospital, Hawally, KWT; 5 Internal Medicine, College of Medicine, University of Lagos, Lagos, NGA; 6 Internal Medicine, Ariana Sabet Hospital, Kabul, AFG; 7 Internal Medicine, Bahria University Medical and Dental College, Karachi, PAK; 8 Internal Medicine, Jinnah Sindh Medical University, Karachi, PAK; 9 Internal Medicine, Liaquat University of Medical and Health Sciences, Jamshoro, PAK

**Keywords:** diabetic complications, diabetic nephropathy, diabetic retinopathy, omentin-1, diabetes mellitus

## Abstract

Introduction: Plasma omentin-1 is a relatively new biomarker that has anti-diabetogenic and its level is inversely related with insulin resistance and diabetes mellitus, thus, increasing the risk of diabetic complications. In this study, we will determine the association between serum plasma omentin-1 levels and diabetes and its complications.

Methods: This case control study was conducted from July 2020 to August 2021 in the internal medicine unit of a tertiary care hospital in Pakistan. There was a total of three groups, each comprising 250 patients. One group was type 2 diabetic patients with complications, the second group was type 2 diabetic patients without complications, and the third group was the control group, i.e. participants without type 2 diabetes. Their plasma omentin-1 levels were compared.

Results: Patients with diabetic complications had significantly lower serum omentin levels than patients without complications (59.01 ± 9.21 ug/dL vs. 75.72 ± 14.11 ug/L; p-value: <0.0001). Patients with diabetes had significantly lower serum omentin levels in comparison to patients without diabetes (75.72 ± 14.11 ug/dL vs. 90.12 ± 17.89 ug/L; p-value: <0.0001).

Conclusion: Based on our study, plasma omentin-1 is negatively associated with diabetes and its complications. Therefore, adequate levels of omentin-1 are needed in order to prevent diabetic complications.

## Introduction

The altered fat distribution, dysfunction and inflammation of adipose tissue, and the production of adipocytokines are thought to contribute to insulin resistance and type 2 diabetes [1]. Long-standing diabetes causes vascular complications which is a major reason for mortality worldwide. Several pathological mechanisms lead to the development of microvascular and macrovascular complications of diabetes.

Visceral adipose tissue, an active endocrine organ, is a source of various adipokines, including leptin, adiponectin, visfatin, tumor necrosis factor-alpha (TNF-α), and interleukin-6 (IL-6). These are the bioactive mediators which decrease inflammation and regulate glucose metabolism by enhancing insulin-mediated glucose transport [2-3]. The anti-inflammatory effect of adipokines occurs through inhibition of TNF-α induced superoxide production which plays a role in maintaining the health of vascular smooth muscle cells [1, 3]. They have variable effects on carbohydrate and lipid metabolism as well [2, 4]. Omentin, a newly identified adipokine with 313 amino acids, encoded by a gene present in chromosomal region 1q22-q23, is related to type 2 diabetes [3, 5]. There are two highly homologous isoforms of omentin, omentin-1 and omentin-2; the former is the major circulating form in human plasma [6]. Studies have found that plasma omentin-1 has anti-diabetogenic and anti-atherogenic properties, whereas its levels are directly related to serum adiponectin and high-density lipoprotein-cholesterol (HDL-C) levels [1] and inversely related with insulin resistance (IR), hyperlipidemia, obesity, and diabetes mellitus [7], thus, increasing the risk of diabetic complications. In this study, we aim to study the correlation between plasma omentin and complications of diabetes.

## Materials and methods

This case control study was conducted from July 2020 to August 2021 in the internal medicine unit of a tertiary care hospital in Pakistan. Informed consent was obtained and a total of 750 participants were enrolled in the study, which were further divided into three equal groups. The study group included 250, already diagnosed, type 2 diabetic patients who had documented evidence of diabetic retinopathy, diabetic peripheral neuropathy, and/or diabetic nephropathy. Retinopathy was confirmed via fundoscopy by the ophthalmologist, nephropathy was diagnosed by urine analysis, and peripheral neuropathy was diagnosed by biothesiometry. Another 250 diabetic patients without complications, adjusted for age, sex, and duration of diabetes, were enrolled in the study. Lastly, another group of 250 patients without the diagnosis of diabetes were included in the study. Ethical review board approval was taken from Kabul University of Medical Sciences (IRB-Notice-112). Participants were explained the entire protocol.

After the process of enrolment, demographics such as age, gender, duration of diabetes, and co-morbidities were noted in a self-structured questionnaire. The venous blood sample was taken from the cubital vein in the morning and sent to the laboratory to assess the level of plasma omentin-1. Plasma omentin was assessed using enzyme-linked immunosorbent assay.

The statistical software “Statistical Packages for Social Sciences (SPSS) v. 23.0 (IBM Corporation, Armonk, New York)” was used to analyze the data. Mean and standard deviation represented numerical data such as age, duration of diabetes, and plasma omentin-1 levels. Chi-square was applied to compare categorical data such as co-morbidities. A p-value of less than 0.05 meant that there is a difference between the case and control group and the null hypothesis is void.

## Results

No significant difference was found in duration diabetes in diabetic patients with and without complications. The demographics and risk factor profile were comparable in all groups and there was no significant difference between them (Table 1).

**Table 1:** Characteristics of the study participants. BMI, body mass index; CAD, coronary artery disease, kg/m2, kilograms per square meters; NA, not applicable; NS, nonsignificant; SD, standard deviation

Characteristics	Diabetes with complications (n=250)	Diabetes without complications (n=250)	Control group (n=250)	p-value
Age in years (mean ± SD)	53 ± 10	53 ± 11	54 ± 11	NS
Duration of diabetes (mean ± SD)	6 ± 2	7 ± 2	NA	NS
Male (%)	131 (52.4)	129 (51.6)	133 (53.2)	NS
Hypertension (%)	171 (68.4)	174 (69.6)	170 (68)	NS
Smoking (%)	61 (24.4)	59( 23.6)	62 (24.8)	NS
BMI more than 25 kg/m^2^ (%)	101 (40.4)	99 (39.6)	105 (42)	NS

Patients with diabetic complications had significantly lower serum omentin levels than patients without complications (59.01 ± 9.21 ug/dL vs. 75.72 ± 14.11 ug/L; p-value: <0.0001). Patients with diabetes had significantly lower serum omentin levels in comparison to patients without diabetes (75.72 ± 14.11 ug/dL vs. 90.12 ± 17.89 ug/L; p-value: <0.0001) (Table 2).

**Table 2:** Comparison of omentin levels in study and control groups. SD, standard deviation

Omentin-1 levels (mean ± SD) (ng/dL)	Diabetes with complications (n= 250)	Diabetes without complications (n= 250)	p-value	Control group (n= 250)	p-value
Total omentin-1	59.01 ± 9.21	75.72 ± 14.11	<0.0001	90.12 ± 17.89	<0.0001
Male	59.81 ± 9.01	76.12 ± 13.98	<0.0001	89.92 ± 16.92	<0.0001
Female	57.99 ± 9.78	75.01 ± 14.02	<0.0001	91.99 ± 18.02	<0.0001

## Discussion

According to our findings, plasma omentin-1 levels are inversely related to diabetic complications. There is very limited information about the relationship between plasma omentin-1 and diabetic complications. Biscetti et al. found that omentin-1 levels in diabetic patients with peripheral artery disease (PAD) are statistically lower than in diabetic patients without PAD [5]. Furthermore, the study by Biscetti et al. indicated that omentin-1 is not only engaged in energy balancing but also has a protective impact on diabetic patients due to its involvement in lipid metabolism and inflammation, both of which contribute to diabetes-related vascular problems [5]. Diabetic patients had considerably lower plasma omentin-1 levels than the overall population in our investigation. Overall, a large number of recent research examining the relationship between omentin-1 concentrations and diabetes showed conflicting results. Abdelraouf Korany et al. also found omentin-1 concentrations to be lower in diabetic individuals [4]. However, on the other hand, Hayashi et al. discovered that diabetic patients had higher omentin-1 concentrations than controls [8].

Adipokines and visceral adipose tissues have received a great deal of interest in recent years. Adipokines are a group of cytokines produced by adipose tissue that are responsible for a variety of harmful and protective actions [9-10]. Omentin, a novel adipokine that is mostly expressed in visceral adipose tissue has been proposed as a biomarker of metabolic disease [11]. Serum omentin levels were previously linked to metabolic risk variables in a negative way. Omentin has also been shown to have anti-inflammatory and insulin-sensitizing properties [12].

Inflammation is a key factor in the development of atherosclerotic plaque, especially in diabetic patients. The onset and progression of atherosclerotic disease might result in a state of latent chronic inflammation. This inflammation is triggered by a variety of routes, mediated by hyperglycemia, and is influenced by oxidation-reduction state and inflammatory cytokine release [13-15]. Through the production of cytokines that directly or indirectly enhance inflammatory pathways, adipose tissue is a major source of inflammation [16].

Diabetes and its complications are associated with significant morbidity and mortality. It is important to identify any biomarker that may predict complications or be a potential treatment opportunity. Our study adds to very limited data available related to the association between complications of diabetes and plasma omentin-1 level. However, there are various limitations as well. Firstly, since the study was case control, the definite association between plasma omentin-1 levels and complications of diabetes could not be established. Secondly, all the participants were taken from a single institute, the sample size was less diverse. Further large cohort studies to determine the exact mechanism of action of plasma omentin-1 in diabetes need to be conducted.

## Conclusions

Our study indicates that omentin-1 is negatively associated with diabetes and its complications and it has a protective role in diabetic patients. Therefore, adequate levels of omentin-1 are needed in order to prevent diabetic complications. Plasma omentin-1 levels correlate with disease severity. Hence, its level can be used by clinicians for the early diagnosis and management of diabetes. It can be a potential biomarker to predict the development of complications in diabetic patients.
